# Reconstruction and prediction of viral disease epidemics

**DOI:** 10.1017/S0950268818002881

**Published:** 2018-11-05

**Authors:** M. U. G. Kraemer, D. A. T. Cummings, S. Funk, R. C. Reiner, N. R. Faria, O. G. Pybus, S. Cauchemez

**Affiliations:** 1 Harvard Medical School, Harvard University, Boston, MA, USA; 2 Computational Epidemiology Lab, Boston Children's Hospital, Boston, MA, USA; 3 Department of Zoology, University of Oxford, Oxford, UK; 4 Department of Biology, University of Florida, Gainesville, Florida, USA; 5 Emerging Pathogens Institute, University of Florida, Gainesville, Florida, USA; 6 Department of Infectious Disease Epidemiology, London School of Hygiene and Tropical Medicine, London, UK; 7 Centre for the Mathematical Modelling of Infectious Diseases, London School of Hygiene & Tropical Medicine, London, UK; 8 Institute for Health Metrics and Evaluation, University of Washington, Seattle, USA; 9 Mathematical Modelling of Infectious Diseases and Center of Bioinformatics, Biostatistics and Integrative Biology, Institut Pasteur, Paris, France; 10 CNRS UMR2000: Génomique évolutive, modélisation et santé, Paris, France

**Keywords:** Geographic spread, real-time prediction, viral epidemics

## Abstract

A growing number of infectious pathogens are spreading among geographic regions. Some pathogens that were previously not considered to pose a general threat to human health have emerged at regional and global scales, such as Zika and Ebola Virus Disease. Other pathogens, such as yellow fever virus, were previously thought to be under control but have recently re-emerged, causing new challenges to public health organisations. A wide array of new modelling techniques, aided by increased computing capabilities, novel diagnostic tools, and the increased speed and availability of genomic sequencing allow researchers to identify new pathogens more rapidly, assess the likelihood of geographic spread, and quantify the speed of human-to-human transmission. Despite some initial successes in predicting the spread of acute viral infections, the practicalities and sustainability of such approaches will need to be evaluated in the context of public health responses.

## Introduction

Infectious disease outbreaks pose a significant threat to human health. The frequency of such outbreaks is thought to have increased over the past decade. For example, quickly after an epidemic of Ebola virus affected Guinea, Sierra Leone, and Liberia in 2013–2016 [1], chikungunya virus (CHIKV) caused an extensive international epidemic in the Americas and beyond, and was quickly followed by Zika virus (ZIKV) emergence. To date, there have been more than 500 000 confirmed or probable cases of ZIKV but the true number of cases remains unknown [2]. Yellow fever (YF), a vaccine-preventable disease, recently posed major public health problems. In 2015–2016, the largest YF outbreak since the 1980s was observed in Angola and the Democratic Republic of the Congo, causing 962 confirmed cases and 393 deaths [3]. YF also poses an ongoing public health risk to large, urban and under-vaccinated populations in the coastal areas of southern Brazil, a country that successfully eradicated YF in the 1950s and 1960s [3–5]. Examples of other emerging pathogens that have caused international health security concerns include the severe acute respiratory syndrome (SARS) virus and the Middle East Respiratory Syndrome Coronavirus (MERS-Cov) [6–9]. This list extends to other pathogens such as influenza, Nipah and henipaviral diseases, and Lassa fever [10]. These examples show the continued risks that infectious diseases pose and highlight the challenges of large international outbreaks to epidemic planning and response.

During emerging infectious disease outbreaks, empirical information and mathematical modelling techniques are now commonly used to characterise and predict the spatio-temporal dynamics of the spread of pathogens. Such analyses may help policymakers to evaluate the threat to public health, determine the resources required to reduce disease burden, and guide disease surveillance efforts and the deployment of interventions.

In the last decade, our ability to perform such assessments has been improved by advances in a number of disciplines, including digital disease surveillance [11], environmental modelling [12, 13], genomics [14] and mathematical modelling [15]. For example, environmental variables such as rainfall and precipitation [13, 16–22] can be used to better understand the landscape within which the disease may be transmitted, and detailed transmission data from a small sampled population can be extrapolated to larger, un-surveyed areas [23]. Attempts have been made to illustrate the spatial structure of epidemics mainly using human movement data [24–27], to provide mechanistic insights in how the disease may disperse locally [3, 28, 29] or how effective reactive vaccination campaigns may be [30–32]. There are continued efforts to reconstruct epidemic dynamics using information derived from pathogen genomic data, which contain unique information about the history of transmission [2, 33–35]. Although each of these disciplines has an established relationship to disease prevention and control, the benefits of integrating them into a unified framework have yet to be fully achieved.

Here we describe the common applications and models used to predict acute viral diseases and discuss the current challenges and limitations. We then outline the advantages of integrating disparate data sources to advance our understanding of epidemic spread. We discuss how such research has been used in recent outbreaks and outline shortcomings that may be addressed in the future.

## Reconstruction of transmission pathways using genomic data

**Fig. 1.**
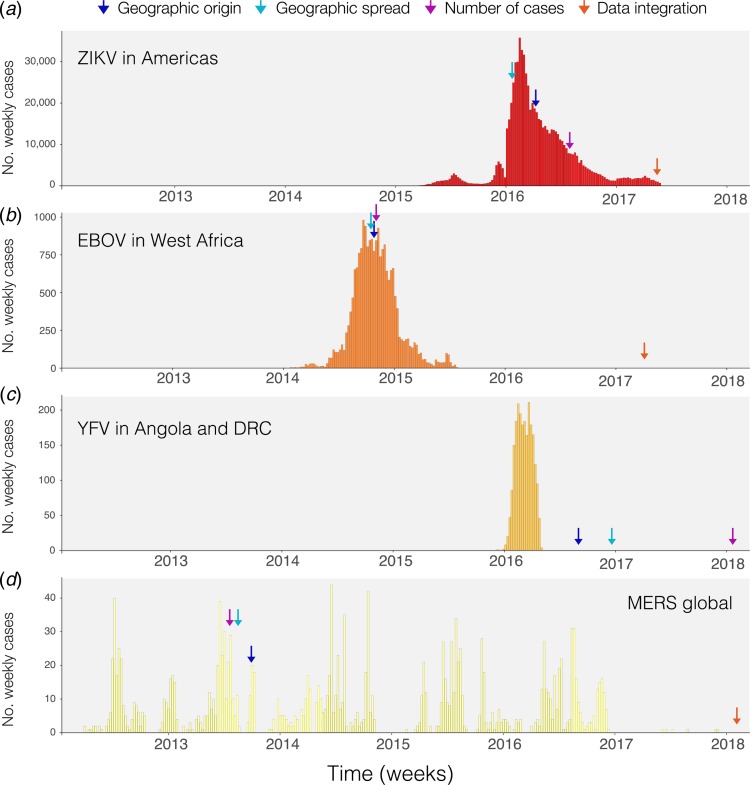
Timing of publications addressing key questions during outbreaks. Blue shows the first peer-reviewed publication identifying the geographic origin of the outbreak, green shows the date predictions about geographic spread are published, purple shows the date when predictions of numbers of cases are made and red indicates the date when work on the integration of geographic, genomic and epidemiological data was published. (a) Shows weekly cases of the 2014–2017 Zika virus epidemic in the Americas using data from [33, 38] and the Pan American Health Organization (PAHO) available from https://github.com/andersen-lab/Zika-cases-PAHO. (b) Shows weekly cases from the West African Ebola epidemic published by the World Health Organization (WHO). (c) Shows weekly cases of the 2015–2016 Yellow fever epidemic in Angola and the Democratic Republic of Congo, published by WHO [40]. (d) Shows weekly cases from 2012 to 2017 Middle Eastern Respiratory Syndrome outbreak available from https://github.com/rambaut/MERS-Cases.

Phylogenetic and phylodynamic tools are increasingly being used to infer a range of outbreak properties [36]. Common spatio-temporal analyses of pathogen genomes focus on mapping and predicting virus lineage exchange among locations, with the underlying aim of reconstructing the pathways of disease introduction and spread, albeit at a coarse spatial resolution, and often retrospectively [2, 8, 33, 35, 37, 38]. An additional feature that can be inferred from genomic data is the timing of individual *founder introductions* [39]. Blue arrows in Fig. 1 indicate the time when the first report was published inferring the likely geographic origin of four major international infectious disease outbreaks. Phylogenetic tools can help to characterise the number of introductions that lead to disease transmission in a new location [41], quantify the risk of cross-species transmission [42], and infer ecological drivers of transmission [43, 44]. Genome-derived estimates have been compared qualitatively to those from epidemiological data, but formal model-based integration of both data sources are rare [45, 46]. In principle, pairing genomic information with epidemiological inference should enable us to quantify the number of cases missed in each location and help to estimate parameters such as the basic reproductive number and doubling time of the epidemic, as done for ZIKV at the tail end of the epidemic (Fig. 1a) [46–48]. A common limitation when genetic data are used is the absence of a rigorous and formal sampling scheme. In many instances, genomic sampling is affected by convenience and expedience and may not reflect underlying incidence, although this can be improved post-hoc in large data sets via sub-sampling using, for example continuous phylogenetic inference [49–51]. Strong sampling biases may affect estimates of the arrival time of a pathogen and its pathways of dissemination among locations [33].

## Prediction of disease spread using spatial information

Static disease mapping is a powerful tool to visualise and defines the landscape within which transmission occurs, based on ecological drivers of transmission [17, 18, 22]. When combined with global data on human travel and mobility, it can be used to understand the global dynamic risk surface of infectious disease, especially when there are strong ecological determinants of transmission, as there are for the vector-borne diseases Zika, dengue, chikungunya and YF [27, 52]. Publication of reports that estimate geographic spread for the diseases in Fig. 1 are indicated by green arrows. The global epidemic history of Zika, for example, remains poorly understood. The challenge to accurately reconstruct the epidemic pathway of the virus is further complicated by its relatively unspecific clinical presentation. This may explain why the initial studies that aimed to understand the geographic origin of the Zika epidemic in the Americas were published relatively late into the epidemic (>1 year, Fig. 1a). For the other major outbreaks highlighted in Fig. 1, estimates of the geographic origin were documented between 6 and 8 months after the first reports of human cases (Fig. 1b–d; Table 1). However, given the underlying ecological determinants of transmission that restrict the reproduction of the virus in the mosquito vector species, large areas can be excluded from the risk of local virus transmission. When overlaying information on the reported presence of Zika cases *vs.* the underlying ecological risk map, surveillance gaps may be identified [19, 27]. Areas where there is a mismatch in the predicted presence and reported presence (i.e. cases detected) should be targeted for active surveillance.

**Table 1.** Key dates and publications describing the geographic origin and spread of four major international outbreaks prediction of the expected number of cases, and integration of geographical, epidemiological and genetic data

Characteristic of outbreak	Date published (online)	Citation
Zika virus outbreak in the Americas (2014–2017)		
Geographic origin	24 March 2016	Faria *et al*., 2016 [2]
Geographic spread	14 January 2016	Bogoch *et al*., 2016 [52]
Number of cases	25 July 2016	Perkins *et al*., 2016 [23]
Integration of genomic/epidemiological/geographical data	17 May 2016	Grubaugh *et al*., 2017 [33] and Faria *et al*., 2017[38]
Ebola virus disease outbreak in West Africa (2013–2016)		
Geographic origin	12 September 2016	Gire *et al*., 2014 [53]
Geographic spread	2 September 2014	Gomes *et al*., 2014 [54]
Number of cases	8 September 2016	Fisman *et al*., 2014 [55]
Integration of genomic/epidemiological/geographical data	12 April 2017	Dudas *et al*., 2017 [35]
Yellow fever outbreak in Central Africa (2015–2016)		
Geographic origin	20 September 2016	Grobbelaar *et al*., 2016 [56]
Geographic spread	22 December 2016	Kraemer *et al*., 2016 [3]
Number of cases	16 January 2018	Zhao *et al*., 2018 [57]
Integration of genomic/epidemiological/geographical data	NA	NA
Middle East Respiratory Syndrome outbreak (2012–2017)		
Geographic origin	20 September 2013	Cotten *et al*., 2013 [58]
Geographic spread	17 July 2013	Khan *et al*., 2013 [59]
Number of cases	5 July 2013	Breban *et al*., 2013 [60]
Integration of genomic/epidemiological/geographical data	16 January 2018	Dudas *et al*., 2018 [42]

The spatial spread process of new pathogens, however, is not only determined by the underlying ecological determinants in each location but also by the dynamic nature of importation, often driven by human movements [61]. Spatial models that take into account the patterns of human spread and mobility may, therefore, improve our ability to characterise and anticipate spatial expansion. Different models have been proposed to predict the geographic spread of epidemics but rarely have they been used in real time during the course of an epidemic [3, 62] (Fig. 1). For example, during the YF outbreak in Angola and the Democratic Republic of the Congo, estimates of geographic spread to provinces outside Luanda, the capital of Angola, were published >6 months after the last cases were reported (Fig. 1c). Such information could guide public health institutions to decide where and when to implement surveillance and control programs [27]. More work, however, is needed to dynamically map the spread of infectious diseases and to extract meaningful and interpretable quantities for public health practitioners.

## Characterising disease dynamics and transmission clusters

In parallel to these efforts to model the spread of pathogens at a meta-population level (e.g. between cities, regions, countries or continents), we also need to better understand transmission dynamics at a much more granular level and assess the characteristics of the inter-human transmission. While historically, the potential for inter-human transmission has often been summarised with a single statistic; the reproduction number *R* (i.e. the average number of secondary infections generated by a case). However, it has long been recognised that it is also essential to assess heterogeneities in individual *R* values, since the presence of super-spreading events may have a major impact on the risk of emergence and our ability to control outbreaks [63]. This was exemplified in a large MERS-CoV outbreak in South Korea in 2015 in which only a small number of cases were responsible for the majority of infections [64, 65]. Other factors that may drive the spatial differences in the reproductive number are ecological (population density, climatic factors, or others) and can now be readily incorporated in transmission models [66].

Ideally, these assessments should be performed on detailed data documenting chains of transmission, as such data can provide precise quantification of the transmission potential and the impact of targeted interventions in different settings and over time, and allow testing specific hypotheses about the transmission process (e.g. what is the contribution of re-introductions to the overall dynamic?) [67]. However, such data are rarely available as it is difficult to identify the source of infection for most pathogens. As a result, sophisticated statistical techniques are often required to reconstruct chains of transmission and estimate transmission parameters from more limited data that may include: (i) in the context of zoonoses, the size of human clusters [68–70] or the proportion of surveillance cases that reported a contact with the natural reservoir [71], (ii) the growth rate in the case count [72–75], (iii) partial data on chains of transmission [76], or (iv) outbreak data where the timing of symptom onsets and location of cases are recorded in small communities such as households [77–80], schools [81] or villages [82]. In cases of high-density sampling, genomic data can help to reconstruct transmission chains [83].

## Disease outbreak modelling

Mechanistic models of infectious disease dynamics can be used to make predictions about the future course of an outbreak within a given location [84]. Increasingly, such models are being used in real time, such that predictions are updated every time a new data point becomes available [85, 86]. Some other applications track pathogen evolution over time as data become available [87]. However, the perceived ability of such models to successfully or unsuccessfully make ‘correct’ predictions can generate considerable controversy [88, 89]. There are few studies that systematically investigate forecasting accuracy and its relationship to the length of time that is being predicted and to the quantity and quality of data available [90, 91]. Other examples are forecasting challenges for ongoing epidemics such as CHIKV in the Americas (https://www.darpa.mil/news-events/2014-08-15), EVD in West Africa [92] and seasonal influenza [85, 93], designed and initiated by funding agencies and public health governments. This is an important area for future research.

## Integrating data sources to better characterise infectious disease dynamics

There are clear benefits to combining information from different data sources in order to better predict viral epidemic spread. Previous work most commonly presents estimates from different sources side-by-side, for example, estimates of the epidemic reproductive number derived from genomic *vs.* epidemiological data [46]. Such comparisons are important to assess the consistency of data sources and may help to derive new hypotheses. Spurred by technological innovation such as portable sequencing using the MinION device (Oxford Nanopore Technologies, Oxford, UK) [94] and by interdisciplinary collaborations during disease outbreaks, researchers have started to work to combine three types of transmission data: spatial, genomic and epidemiological which have now been published for three of the four major outbreaks we considered here (Fig. 1, red arrows) [33, 38, 41].

For example, such interdisciplinary work helped to identify the introduction of Zika into the Americas [2], investigated the main drivers of transmission of ZIKV through climatic suitability of its mosquito vectors [25] and tried to extrapolate how many people had been infected with the virus [23, 92, 93]. In the context of phylogenetic analyses, environmental and other spatial data may be helpful in reconstructing the drivers of transmission and spread using, for example, information on the reservoir or host movements [35, 95]. In turn, phylogenetic information may complement epidemiological analysis by providing more evidence on the transmission routes that are common in an outbreak [96]. This may be particularly useful for diseases that have a highly structured transmission dynamic, such as MERS or SARS, where a small number of people are responsible for the majority of secondary cases [63, 97], transmission from the animal reservoir is frequent, or importation drives locally observed epidemics [33]. One common assumption in many epidemiological models is that it is equally likely for people to meet and infect others living in the same location and that population immunity is proportional to the demographic structure [98]. Hence, observed cases are often assumed to arise from other cases that are reported locally as long as they are consistent with the generation time of the disease. However, a well-connected location can, in principle, accrue a large number of incident cases through recurring introductions from elsewhere, rather than via local transmission [33]. These results can have large implications for surveillance and control, as different competing strategies (e.g. limiting importations or eradicating the disease locally) may be considered. While analytical approaches of various degrees of complexity have been proposed to probabilistically reconstruct transmission trees from incomplete outbreak data [73, 81, 97], contact tracing, which can be very labour intensive [67], remains a gold standard information source. This may allow us to is to determine the true distribution of cluster sizes (i.e. the number of subsequent cases resulting from each introduction) but is often only available for a small number of locations. However, using genomic data can help refine the understanding of heterogeneity in transmission but such framework does not yet allow to exactly quantify the fraction of observed cases that are attributable to local transmission versus introduction from elsewhere, or to determine how many importations are responsible for the local incidence, despite its crucial importance for eradication campaigns [42, 100, 101]. In the context of the Zika outbreak in Florida, combining genomic data from the outbreak with epidemiological analysis revealed that the outbreak was driven by a large number of introductions rather than by persistent local transmission. In the recent yellow fever outbreak in southern Brazil, linking epidemiological, spatial and genomic data and techniques could provide insights into the transmission potential and risk of urban transmission [102]. One dataset and analysis alone would have not been strong enough to make such conclusions [33].

Inferences about epidemic processes made using mathematical models rely on a number of assumptions. Geographic modelling approaches, mostly informed by spatial ecology, attempt to fill gaps where no data has been observed, hence inferences may be uncertain, as the underlying ecological process may be poorly understood and dynamical aspects of the invasion process are ignored. These deficiencies can be ameliorated, in part, by adding virus genome data that contain information about past transmission and invasion patterns [103]. However, due to incomplete and poor sampling (as discussed above), genomic data alone may provide an incomplete picture of the timing of viral introduction and spread among locations. This, in turn, can be supported by the addition of epidemiological time series of reported cases and serological information about population immunity [104, 105]. Despite this, building a joint inference framework that combines all available data sources and which characterises observation and sampling processes correctly is a daunting task. However, we are entering a period where the data for this task are becoming available in a timely fashion but need to ensure that results are communicated as soon as they are generated in order to avoid delays. Initial successes have already led to important advancements in epidemic control and should progress to a tool-kit for guiding public health, hopefully available in real time for future epidemics.
